# Anaplastic large-cell lymphoma with atypical chromosomal translocation t(2;5) and hypophyseal tumor

**DOI:** 10.3109/03009734.2010.500061

**Published:** 2010-10-27

**Authors:** Lei Wang, Yin Tong, Zhimei Chen, Weilai Xu, Jie Jin

**Affiliations:** ^1^Department of Hematology, First Affiliated Hospital, College of Medicine, Zhejiang University, HangzhouP. R. China; ^2^Institute of Hematology, First Affiliated Hospital, College of Medicine, Zhejiang University, HangzhouP. R. China

**Keywords:** ALCL, ALK+, chromosomal translocation, hypophyseal tumor

## Abstract

**Abstract:**

Anaplastic large T-cell lymphoma (ALCL) is frequently associated with the t(2;5)(p23;q35). Here, we report a case of ALCL with an atypical translocation of t(2;5)(p24;q13) and other complex translocations. This complex abnormal karyotype may result in chemotherapy resistance and a poor outcome. Interestingly, a hypophyseal tumor was detected simultaneously by magnetic resonance imaging in this case. We think it is probable that the tumor in the hypophysis might be associated with the ALCL.

## A case report

**Figure 1.**
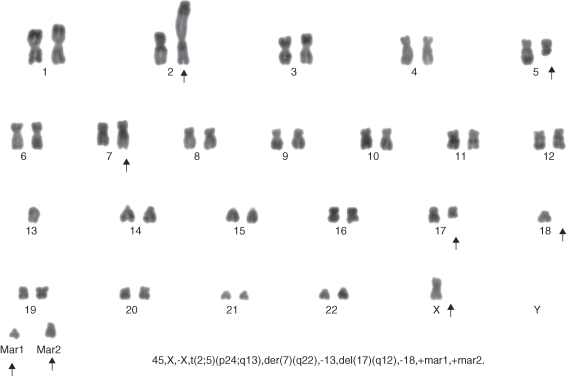
Karyotype: 46, X,-X, t(2;5)(p24;q13), der(7)(q22), -13,del(17)(q12), -18, +mar1, +mar2.

A 19-year-old woman was admitted to our hospital with a 15-day history of lymphadenopathy and fever. Physical examination showed lymphadenophathy and splenomegaly. Hemogram showed hemoglobin (Hb) 93 g/L, white blood cell (WBC) 39 × 10^9^/L, platelets 273 × 10^9^/L, with differential count of lymphocytes 44%, monocytes 8%, eosinophils 2%, neutrophilic granulocytes 40%, and immature cells 6%. The WBC counts rose to 122 × 10^9^/L, and the platelet counts dropped to 16 × 10^9^/L in one week. Bone-marrow aspiration and biopsy revealed hypercellular, predominant myeloid cells showing a full range of maturation. Karyotypic analysis showed: 46, X,-X, t(2;5)(p24;q13), der(7)(q22), del(17)(q12), -18, +1-2mar[3]/46, XX, der(2), t(2;5)(p24;q13), -5, del(17)(q12), -18, +1-2mar[1]/47, idem, +8[1]/46, XY, normal karyotype[5] (Figure 1). Computed tomography (CT) scan of the abdomen and chest showed splenomegaly and lymphadenophathy. She underwent an excisional biopsy of an enlarged inguinal lymph node. The architecture of the lymph node was distorted by cellular proliferation, but sinusoids were still present. The cellular proliferation included small lymphoid cells and large lymphoid cells with round nuclei, admixed with some spindle cells. On immunostaining, the large cells were negative for CD2, CD3, CD5, and CD7, but were positive for CD43. They were CD30+ and ALK+ (nuclear cytoplasmic pattern). A diagnosis of primary systemic anaplastic large-cell lymphoma, ALK+, T-cell-type was made. Analysis of cells from bone-marrow by flow cytometry confirmed the presence of abnormal T-cells. The patient was initially treated with one cycle of VDCP (cyclophosphamide 750 mg/m^2^ on days 1 and 8, liposomal doxorubicin 8 mg/m^2^ on days 1 through 3, vincristine 1.4 mg/m^2^ on days 1 and 8, and prednisone 1 mg/kg/d on days 1 through 14 and 0.5 mg/kg/d on days 15 through 21), after which the enlarged lymph nodes disappeared. But the percentage of lymphocytes was still high, and the patient had fever every day. The repeated analysis by flow cytometry still showed the existence of abnormal T-cells. Another cycle of CHOP (cyclophosphamide 750 mg/m^2^ on day 1, vincristine 1.4 mg/m^2^ on day 1, doxorubicin 50 mg/m^2^ on day 1, and prednisone 100 mg/d on days 1 through 5) was given followed by VP16 (etoposide) plus cytarabine (Ara-C). She began to feel fatigue one week later even though red blood cells were infused several times. Levels of thyroid hormones, including total triiodothyronine (TT3), total thyroxine (TT4), thyrotropic-stimulating hormone (TSH), free triiodothyronine (FT3), and free thyroxine (FT4), were very low. Enhanced magnetic resonance imaging (MRI) of the brain indicated a tumor in the hypophysis. Euthyrox was given, and then she felt much better. Repeated analysis by flow cytometry showed the remarkable decrease of abnormal T-cells. Unfortunately, the patient finally died due to pneumonia one week later.

**Figure 2.**
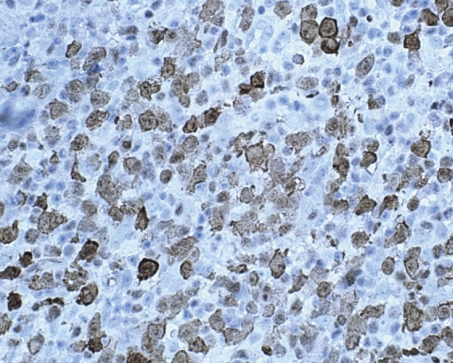
Biopsy of an enlarged inguinal lymph-node. Immunostaining showed the large cells were positive for ALK-1 (×400).

Anaplastic large T-cell lymphoma (ALCL) was first recognized in 1985 by Stein et al. who reported consistent expression of Ki-1 antigen (later designated CD30) with frequent cohesive proliferation of large pleomorphic cells. Two distinct clinical forms of primary ALCL are now recognized: limited to the skin and systemic. It is frequently associated with the t(2;5)(p23;q35), resulting in expression of a fusion gene nucleophosmin-anaplastic lymphoma kinase (NPM-ALK). Variant translocations other than t(2;5) occur in up to 15%–20% of the cases: these include t(1;2)(q21;p23) (chimeric protein TPM3-ALK); inv(2)(p23;q35) (ATIC-ALK); t(2;3)(p23;q21) (TRK-fused gene (TFG)-ALK); t(2;19)(p23;q13.1) (tropomyosin 4 (TPM4)-ALK); t(2;X)(p23;q11-12) (moesin (MSN)-ALK), and a clathrin, heavy chain-like 1 (CLTCL)-ALK fusion transcript typically resulting from a t(2;17)(p23;q23) (1,2). Our patient had atypical translocation of t(2;5)(p24;q13), which has different break points in chromosomes compared with typical t(2;5)(p23;q35). This has not been reported in the literature. The translocation also has association with a receptor tyrosine (ALK) gene since the patient was ALK+ by immunostaining. We do not know which type of ALK fusion gene this translocation generated. But it is sure that the translocation caused activated tyrosine kinase that is oncogenic. Though most investigators consider that ALCL generally behaves as a usually intermediate to high-grade lymphoma, still, comparative studies in diffuse large-cell lymphomas have shown an association between ALK expression and a favorable outcome (3). In a series that included 57 patients with T-cell/null ALCL, the 5-year overall survival rate was 93% in patients who were ALK+ compared with 37% for those who were ALK- (4). However, in multivariate analysis, only high international prognosis index (IPI) and CD56 positivity were independent predictors of poor outcome. In an Italian series, ALK+ patients with intermediate or high IPI score had a 5-year overall survival (OS) rate of only 41% (5). It has been reported that the prognosis of patients with variant translocation is similar to that of patients with the classic t(2;5) (6). But in our patient who had atypical t(2;5) and many other complex translocations, these translocations may also be the predictor of poor outcome.

The majority of ALK+ ALCL cases present as advanced stage (III or IV) systemic disease with generalized lymphadenopathy and extranodal involvement, particularly of the skin and soft tissues, with involvement of the gut and nervous system (CNS) being a rare event (4). Bone-marrow involvement has been initially considered as a rare event in ALCL. When immunohistochemistry with anti-CD30 and anti-epithelial membrane antigen (EMA) was used to study bone-marrow samples, malignant cells could be detected in as many as 43% of cases (7). In the bone-marrow, higher WBC counts and lower platelet counts suggested bone-marrow involvement. But we did not find any malignant cells in the bone-marrow. A leukemoid reaction may explain the leukocytosis but cannot explain the decreased platelet count. The analysis by flow cytometry or immunohistochemistry might help to find abnormal cells and distinguish the leukemoid reaction from bone-marrow involvement. Our patient later complained of fatigue, and enhanced MRI of the brain indicated a hypophyseal tumor. To our knowledge, there are no other reports about ALCL involving the hypophysis. Since the patient did not feel any discomfort before the presence of lymphadenopathy, and she felt obvious fatigue due to low levels of thyroid hormones during therapy, we think it is probable that the tumor in the hypophysis might be associated with ALCL.

The corner-stone of most treatment protocols consists of alkylating agents, anthracyclines, vinca alkaloids, and corticosteroids. The US standard has generally been to treat ALCL with CHOP chemotherapy (8). In the literature, it has been reported that the leukemia-like protocol Italian Association of Pediatric Hematology and Oncology (AIEOP)-92 was found to be an effective treatment for childhood ALCL (9). Our patient had a high percentage of abnormal T-cells in the bone-marrow. We therefore treated her with a leukemia-like protocol (VDCP regimen). The patient responded well at first since the enlarged lymph nodes disappeared. But the abnormal T-cells in the bone-marrow remained, which indicated that these cells might be resistant to VDCP chemotherapy. VP16 and Ara-C may have some effect, but bone-marrow suppression was severe. So, further observations are needed to find the best therapeutic regime for this kind of ALCL.
